# Author Correction: Projecting the future incidence and burden of dengue in Southeast Asia

**DOI:** 10.1038/s41467-023-42378-0

**Published:** 2023-10-16

**Authors:** Felipe J. Colón-González, Rory Gibb, Kamran Khan, Alexander Watts, Rachel Lowe, Oliver J. Brady

**Affiliations:** 1 Department of Infectious Disease Epidemiology, Faculty of Epidemiology and Population Health, London School of Hygiene & Tropical Medicine, London, WC1E 7HT UK; 2 Centre for Mathematical Modelling of Infectious Diseases, London School of Hygiene & Tropical Medicine, London, WC1E 7HT UK; 3 Centre on Climate Change and Planetary Health, London School of Hygiene & Tropical Medicine, London, WC1E 7HT UK; 4 Tyndall Centre for Climate Change Research, School of Environmental Sciences, University of East Anglia, Norwich, NR4 7TJ UK; 5 Data for Science and Health, Wellcome Trust, London, NW1 2BE UK; 6 Department of Medicine, Division of Infectious Diseases, University of Toronto, Toronto, ON M5S 3H2 Canada; 7 BlueDot, Toronto, ON M5J 1A7 Canada; 8 Esri Canada, Toronto, ON M3C 3R8 Canada; 9 Barcelona Supercomputing Center (BSC), Barcelona, 08034 Spain; 10 Catalan Institution for Research and Advanced Studies (ICREA), Barcelona, 08010 Spain

**Keywords:** Viral infection, Environmental impact

Correction to: *Nature Communications* 10.1038/s41467-023-41017-y, published online 06 September 2023

The original version of the Article contained an error in the Methods section in that the first sentence of the subsection ‘Epidemiological dengue data’ contained an incorrect reference to the source of data used in the study and in the Supplementary Table 1 in that incorrect links to the source of the data were provided. This has been corrected in both the PDF and HTML versions of the Article. The HTML has been updated to include a corrected version of the Supplementary Information.

### Supplementary information

Updated Supplementary Information

