# Supplementary material for: Projecting the future incidence and burden of dengue in Southeast Asia
Source: Nat Commun. 2023 Sep 6;14:5439. doi: 10.1038/s41467-023-41017-y (PMC10482941; doi:10.1038/s41467-023-41017-y)
Supplement: Supplementary file 1 — Supplementary information [file 41467_2023_41017_MOESM1_ESM.pdf]

Projecting the future incidence and burden of dengue in Southeast Asia

Felipe J. Colón-González*[1,2,3,4,5], Rory Gibb[1,2], Kamran Khan[6,7], Alexander Watts[7,8], Rachel Lowe[1,2,3,9,10], & Oliver J Brady[1,2]

[1] Department of Infectious Disease Epidemiology, Faculty of Epidemiology and Population Health, London School of Hygiene & Tropical Medicine, London, United Kingdom; [2] Centre for Mathematical Modelling of Infectious Diseases, London School of Hygiene & Tropical Medicine, London, UK; [3] Centre on Climate Change and Planetary Health, London School of Hygiene & Tropical Medicine, London, UK; [4] Tyndall Centre for Climate Change Research, School of Environmental Sciences, University of East Anglia, Norwich, UK; [5] Data for Science and Health, Wellcome Trust, London, United Kingdom; [6] Department of Medicine, Division of Infectious Diseases, University of Toronto, Toronto, Canada [7] BlueDot, Toronto, Canada [8] Esri Canada, Toronto, Canada [9] Barcelona Supercomputing Center, Barcelona, Spain; [10] Catalan Institution for Research and Advanced Studies (ICREA), Barcelona, Spain

Supplementary information

Supplementary Tables

Supplementary Table 1: Sources of epidemiological surveillance data used in the study

Country	Period	Source
Cambodia	Jan 2000 to Dec 2010	doi:10.25337/T7/ptycho.v2.0/KH.38362002
Cambodia	Jan 2011 to Dec 2017	http://cdcmoh.gov.kh/surveillance/camewarn
Indonesia	Jan 2000 to Dec 2010	doi:10.25337/T7/ptycho.v2.0/ID.38362002
Indonesia	Jan 2011 to Dec 2017	https://pusdatin.kemkes.go.id/index.php
Laos	Jan 2002 to Dec 2006	doi:10.25337/T7/ptycho.v2.0/LA.38362002
Laos	Jan 2011 to Dec 2013	https://iris.wpro.who.int/
Malaysia	Jan 2000 to Dec 2010	doi:10.25337/T7/ptycho.v2.0/MY.38362002
Malaysia	Jan 2011 to Dec 2017	https://www.data.gov.my/
Philippines	Jan 2000 to Dec 2010	doi:10.25337/T7/ptycho.v2.0/PH.38362002
Philippines	Jan 2013 to Dec 2017	https://www.doh.gov.ph/statistics
Singapore	Jan 2000 to Dec 2010	doi:10.25337/T7/ptycho.v2.0/SG.38362002
Singapore	Jan 2011 to Dec 2017	https://www.moh.gov.sg/resources-statistics/infectious-disease-statistics
Thailand	Jan 2000 to Dec 2010	doi:10.25337/T7/ptycho.v2.0/TH.38362002
Thailand	Jan 2011 to Dec 2017	https://wesr.doe.moph.go.th
Vietnam	Jan 2000 to Dec 2010	doi:10.25337/T7/ptycho.v2.0/VN.38362002
Vietnam	Jan 2011 to Dec 2017	http://soyte.hatinh.gov.vn

Supplementary Figures

Supplementary Figure 1. Spatiotemporal patterns in the observed number of dengue cases and the mean absolute error.

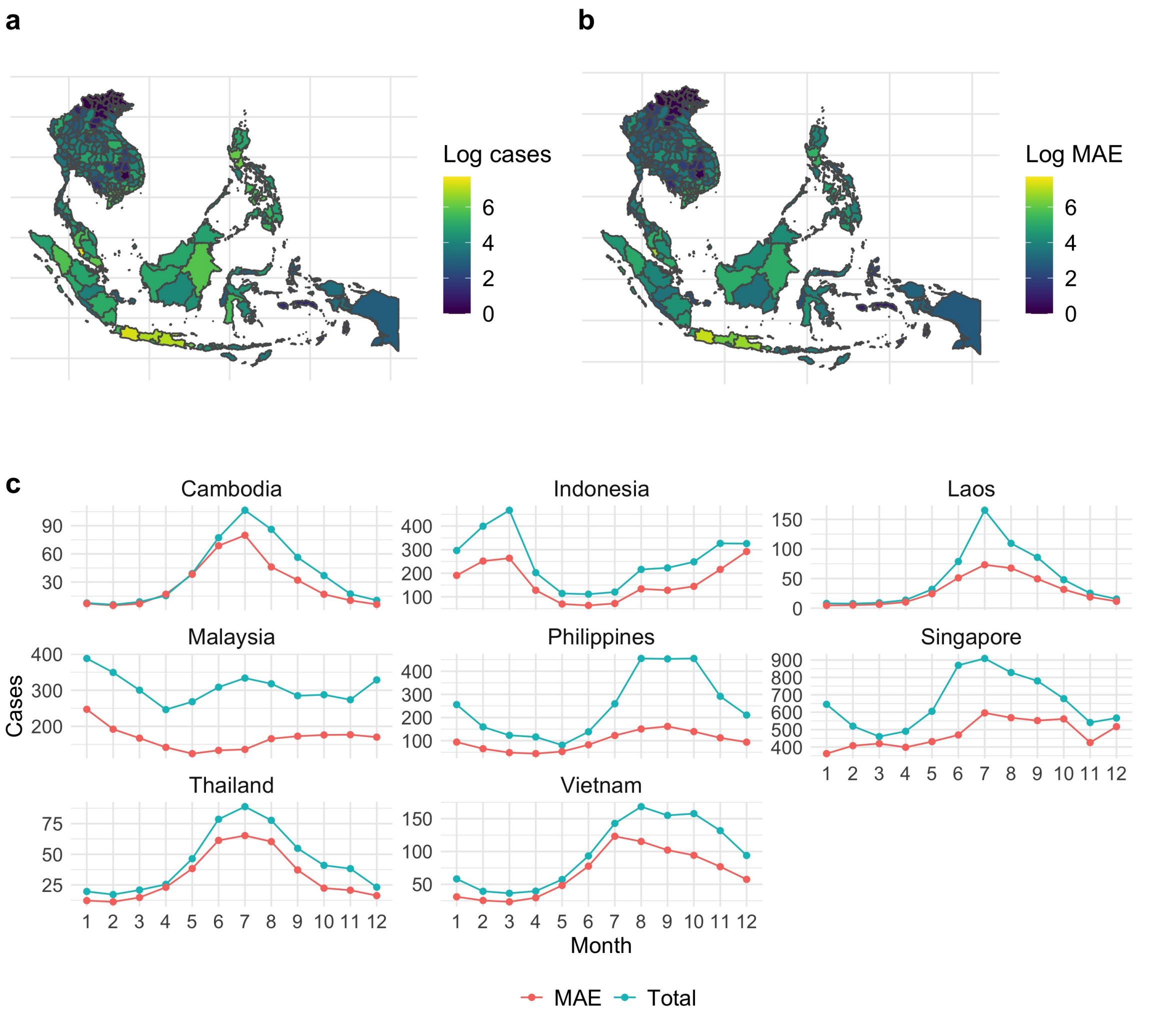

Spatial patterns of a. the mean number of dengue cases and b. median cross-validated mean absolute error (both on a logarithmic scale) per administrative unit across Southeast Asia over the period 2000 to 2017. c. Mean monthly seasonal trends in the mean number of dengue cases (blue) and median cross-validated mean absolute error (red) per country across the historic period 2000 to 2017.

Supplementary Figure 2. Interannual variation in the mean absolute error.

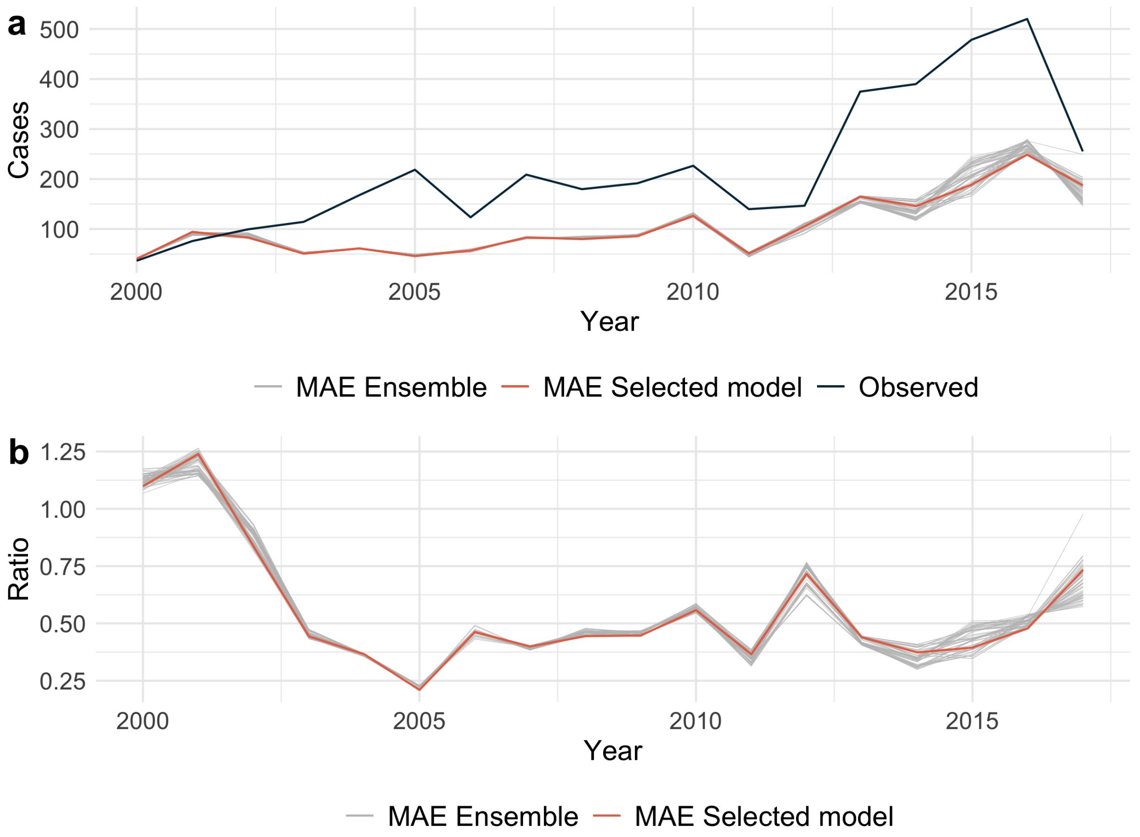

a. Interannual variation in the median cross-validated mean absolute error (MAE) across Southeast Asia over the period 2000 to 2017. b. MAE expressed as a rate of the observed dengue cases. The grey lines indicate the median cross-validated mean absolute error for each of the alternative models explored in the study. The orange line indicates the median cross-validated mean absolute error of the selected model. The blue line indicates the mean number of monthly dengue cases per year.

Supplementary Figure 3. Cross-validated in-sample and out-of-sample correlations between observed and predicted dengue cases.

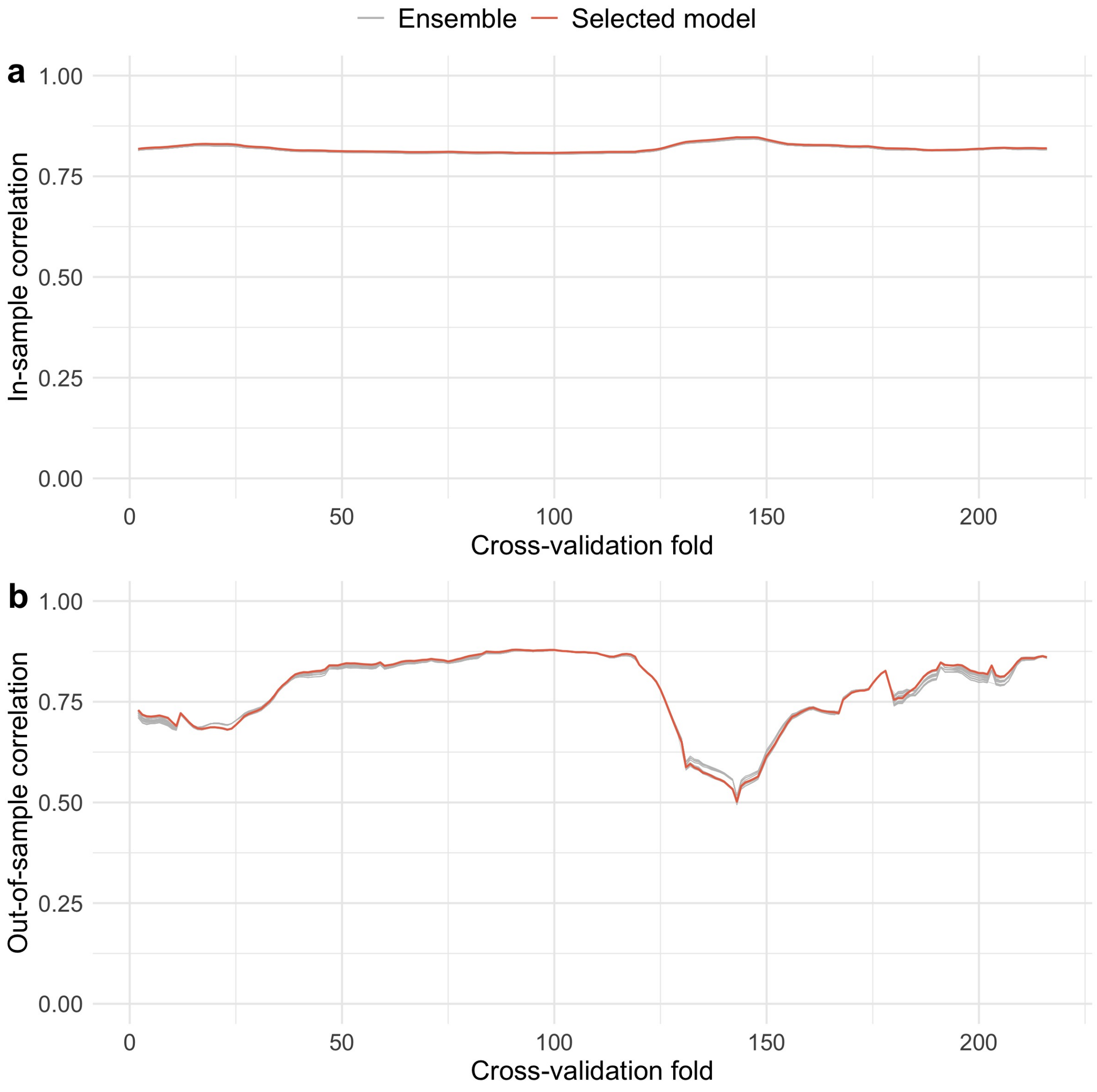

Blocked cross-validation of a. in-sample and b. out-of-sample Spearman rank correlation coefficients between observed and predicted dengue cases over the historic period 2000 to 2017. The grey lines indicate the correlation coefficients for each of the alternative models explored in the study. The orange lines indicate the coefficients of the selected model.

Supplementary Figure 4. Distribution of historical and projected data.

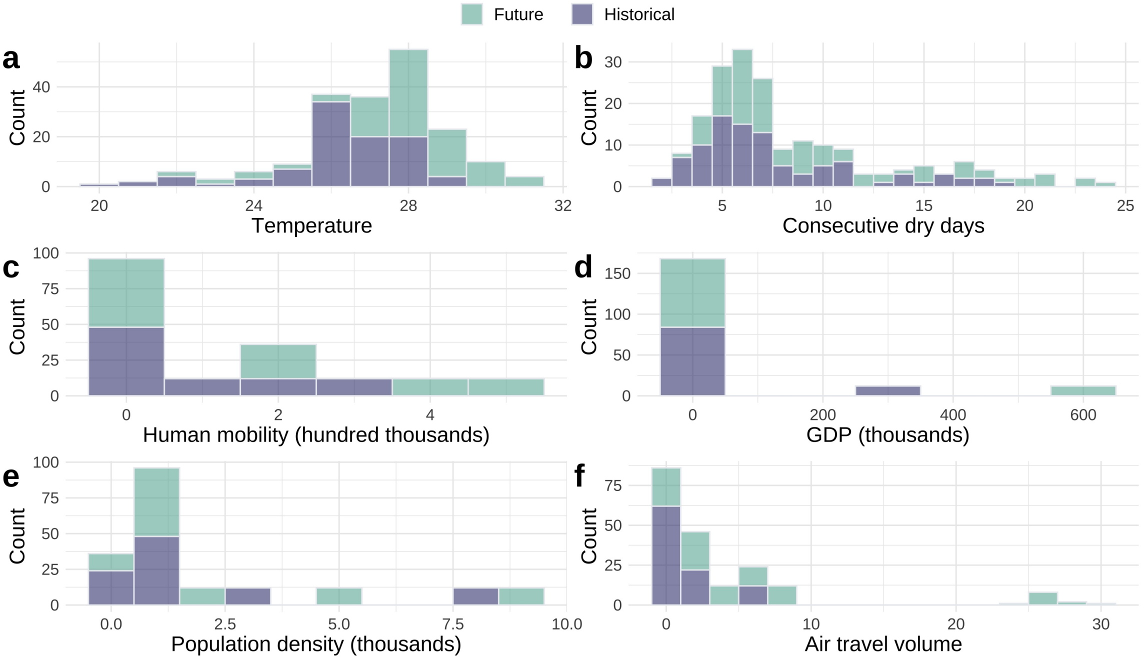

Histograms of the historical (navy blue) and projected (light blue) data for each of the six predictive variables explored in the study.

Supplementary Figure 5. Spatial patterns of population density.

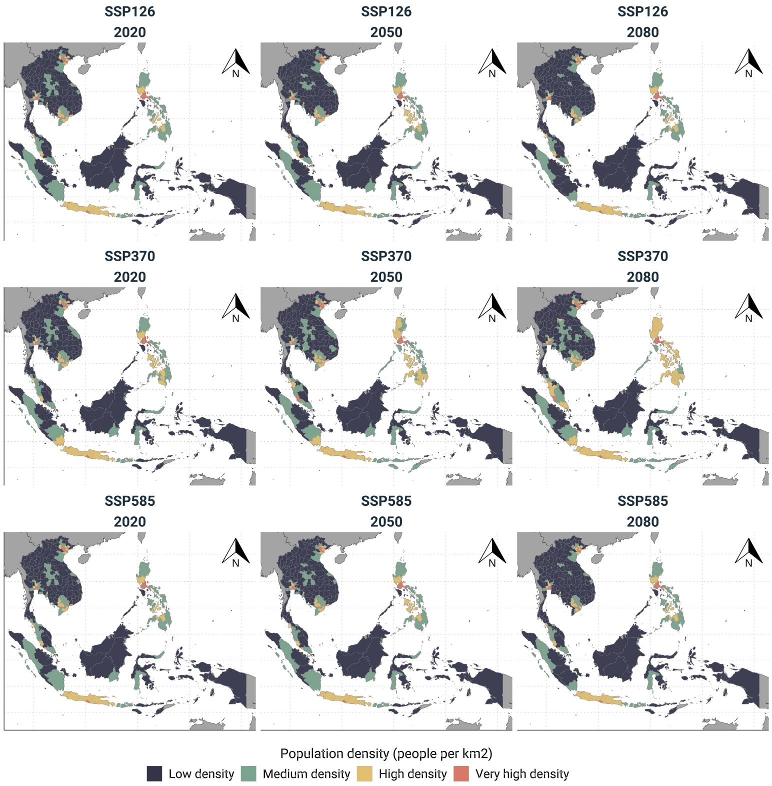

Spatial patterns of population density (people/km^2) across Southeast Asia for the periods 2020, 2050 and 2080, and for each of three scenarios.

Supplementary Figure 6. Sensitivity analysis of the model predictions stratified by country.

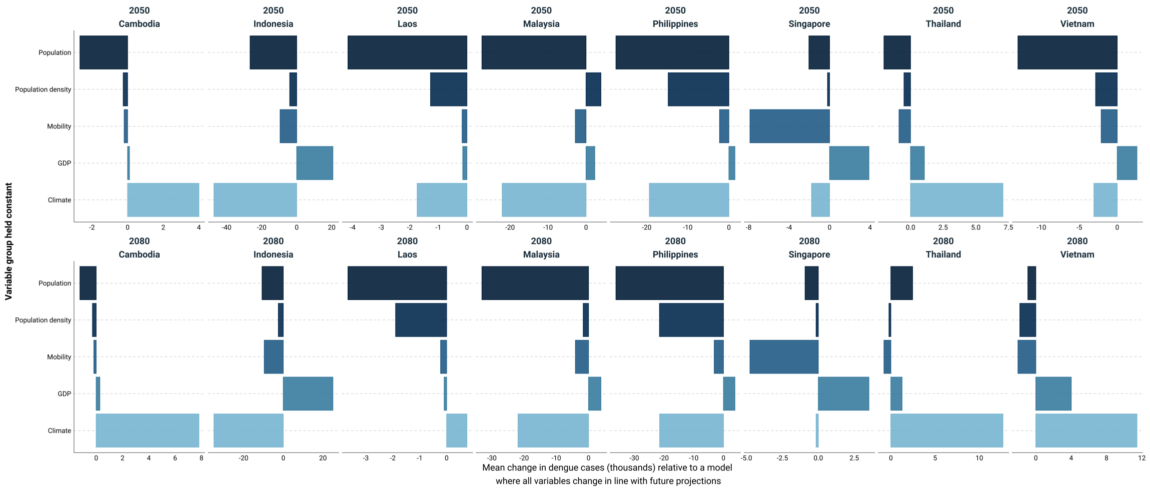

Country-stratified projected changes in mean annual dengue cases (thousands) relative to a model where all variables change in the future, under the assumption that one group of predictors (climate, GDP, human mobility, and population) remains constant at their historical monthly mean values.

Supplementary Figure 7. Yearly random effects.

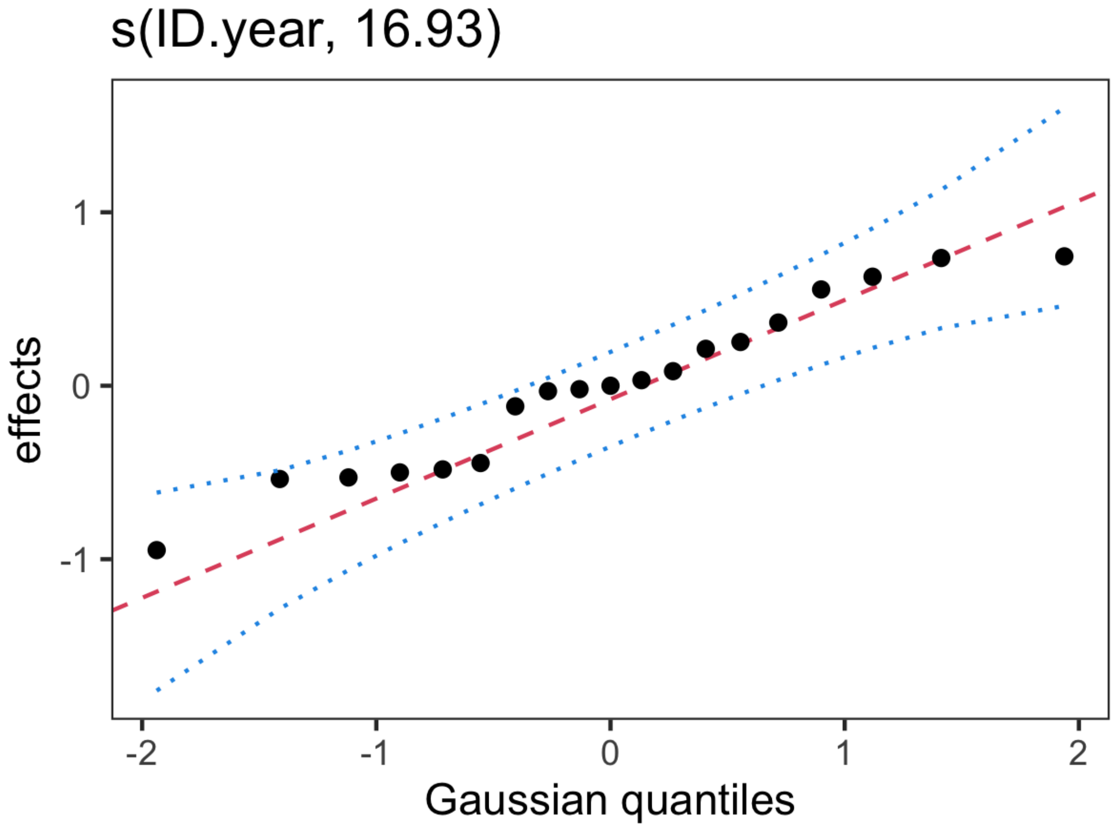

Distribution of yearly random effects included in the final model.
